# Wernicke's Encephalopathy With MRI Findings Despite Coadministration of Thiamine and Glucose

**DOI:** 10.7759/cureus.64192

**Published:** 2024-07-09

**Authors:** Zhaoqian Zhang, Xiao Li, Mei Yang

**Affiliations:** 1 Internal Medicine, St. Luke's Hospital, Chesterfield, USA

**Keywords:** metabolic encephalopathy, altered mental state, mri brain and spine, wernicke-korsakoff syndrome, wernicke encephalopathy

## Abstract

Wernicke's encephalopathy (WE) is a prominent neurologic manifestation of thiamine (vitamin B1) deficiency. While often linked to alcoholism, it can also arise from various causes, including malabsorption, inadequate dietary intake, increased metabolic requirement, and among dialysis patients. Here, we present a case of altered mental status from acute metabolic encephalopathy attributed to sepsis, acute kidney injury (AKI), and hypoglycemia. WE was overlooked in the early hospitalization course due to the daily administration of thiamine. However, the patient's cognitive decline persisted despite the improvement of sepsis and AKI. Subsequent brain MRI revealed thalamic T2 signal intensity changes, suggesting either a past infarction or WE. Implementing an empirical regimen of high-dose thiamine resulted in the patient's rapid cognitive recovery. This therapeutic strategy was integrated into the management of her sepsis and AKI, leading to her full recovery and subsequent hospital discharge without complications.

## Introduction

Wernicke's encephalopathy (WE) is a well-known neurologic manifestation resulting from thiamine (vitamin B1) deficiency. Although WE is commonly associated with chronic alcoholism, it can also emerge from various other causes, such as malabsorption, inadequate dietary intake, increased metabolic requirements, and in patients undergoing dialysis. Diagnosing WE can be particularly challenging due to its nonspecific and variable presentation, which often leads to delayed or missed diagnoses. Early symptoms, such as confusion, ophthalmoplegia, and ataxia, can easily be attributed to other medical conditions, complicating the clinical picture [1-4].

The differential diagnosis of WE includes acute metabolic encephalopathy, stroke, hypoglycemia, and sepsis-associated encephalopathy, each of which requires distinct management strategies. Furthermore, the long-term complications of untreated WE are severe and may include Korsakoff's syndrome, a chronic and debilitating condition characterized by profound memory deficits and confabulation [2-4].

In this report, we present a case of a patient who exhibited altered mental status (AMS) in the context of acute metabolic encephalopathy due to sepsis, acute kidney injury (AKI), and hypoglycemia. Initially, the possibility of WE was overlooked due to the patient receiving daily thiamine supplementation. However, the persistence of cognitive decline despite the resolution of sepsis and AKI prompted further investigation. A subsequent brain MRI revealed thalamic T2 signal intensity changes suggestive of WE. An empirical regimen of high-dose thiamine was administered, leading to rapid cognitive recovery. This case underscores the importance of considering WE in the differential diagnosis of AMS and highlights the need for timely intervention to prevent long-term neurological sequelae [2-6].

## Case presentation

A 58-year-old female with a significant medical history of spinal stenosis status post multiple spinal surgeries and systemic lupus erythematosus presented to the emergency department (ED) with a four-day history of confusion.

Due to the patient living alone, a detailed clinical history could not be obtained. On admission to the ED, the patient exhibited tachycardia (heart rate in the 100s), tachypnea (27 breaths per minute), and a blood pressure of 131/86 mmHg. Physical examination revealed an alert but clearly disoriented patient, who was unable to respond coherently or follow commands. The neurological examination indicated pupils that were equal and reactive, no gaze preference or deviation, no nystagmus, no facial asymmetry, and no nasolabial fold flattening. The patient was able to move all four extremities, but cerebellar function, muscle strength, and sensation could not be accurately assessed due to her altered mental status. Laboratory results (Table 1) suggested acute hypoglycemia, AKI, and a possible infection. She promptly received intravenous sodium bicarbonate, 1 amp of D50 (25 mg dextrose) with 100 mg thiamine, with minimal improvement noted.

**Table 1:** Admission lab results BUN: Blood urea nitrogen; POC: point-of-care

Test	Result	Reference range
pH-ABG	7.08	7.35-7.45
PaCO_2_-ABG (mmHg)	19	35-45
PaO_2_-ABG (mmHg)	74	80-100
HCO_3_-ABG (mmol/L)	5.5	22-26
WBC (x1000/μL)	7	4-11
BUN (mg/dL)	113	20-45
Creatinine (mg/dL)	3.4	0.72-1.25
Procalcitonin (ng/mL)	53.8	0-0.05
Glucose (POC) (mg/dL)	52	70-105

CTA head showed no acute vascular abnormalities. CT chest shows diffuse ground-glass opacities concerning pneumonia. She was immediately given intravenous fluid and broad-spectrum antibiotics and transferred to the medical ICU.

Day 1 (ICU admission)

Upon admission to the ICU, the patient's mental status showed marginal improvement. The patient was in moderate distress, extremely lethargic, and alert and oriented to only person and place (AAO x 2). She was suspected of having acute metabolic encephalopathy due to several factors: sepsis, acute renal failure, severe acidosis, and potential medication effects, as the medication list was unclear. An empirical dose of Narcan yielded no improvement. Cefepime and vancomycin were initiated for possible aspiration pneumonia, as indicated by the chest CT. Keppra 500 mg IV twice daily was started as a precaution against seizures. A point-of-care (POC) glucose recheck revealed a level of 57 mg/dL, prompting the administration of a "banana bag" (vitamin B1 100 mg and folic acid 1 mg) followed by 25 g of D50. The "banana bag" regimen was continued daily thereafter.

Day 2

The patient's cognitive state deteriorated, manifesting as worsening confusion and agitation, despite the recovery of renal function. A continuous EEG was performed and was suggestive of metabolic encephalopathy without any seizure activity. Keppra was discontinued due to the absence of seizure activity. The patient became restless and combative overnight, necessitating sedation with Precedex, Haldol, and Zyprexa.

Day 3

The patient's agitation persisted despite her lab results indicating significant improvement and decent urine output (60 ml/h) (Table 2).

**Table 2:** Lab results on Day 3 The lab results on Day 3 indicated significant improvement with normalized creatinine and a downward trend in BUN levels. BUN: Blood urea nitrogen

Test	Result	Reference range
WBC (x1000/μL)	6	4-11
BUN (mg/dL)	60	20-45
Creatinine (mg/dL)	0.76	0.72-1.25

**Figure 1:**
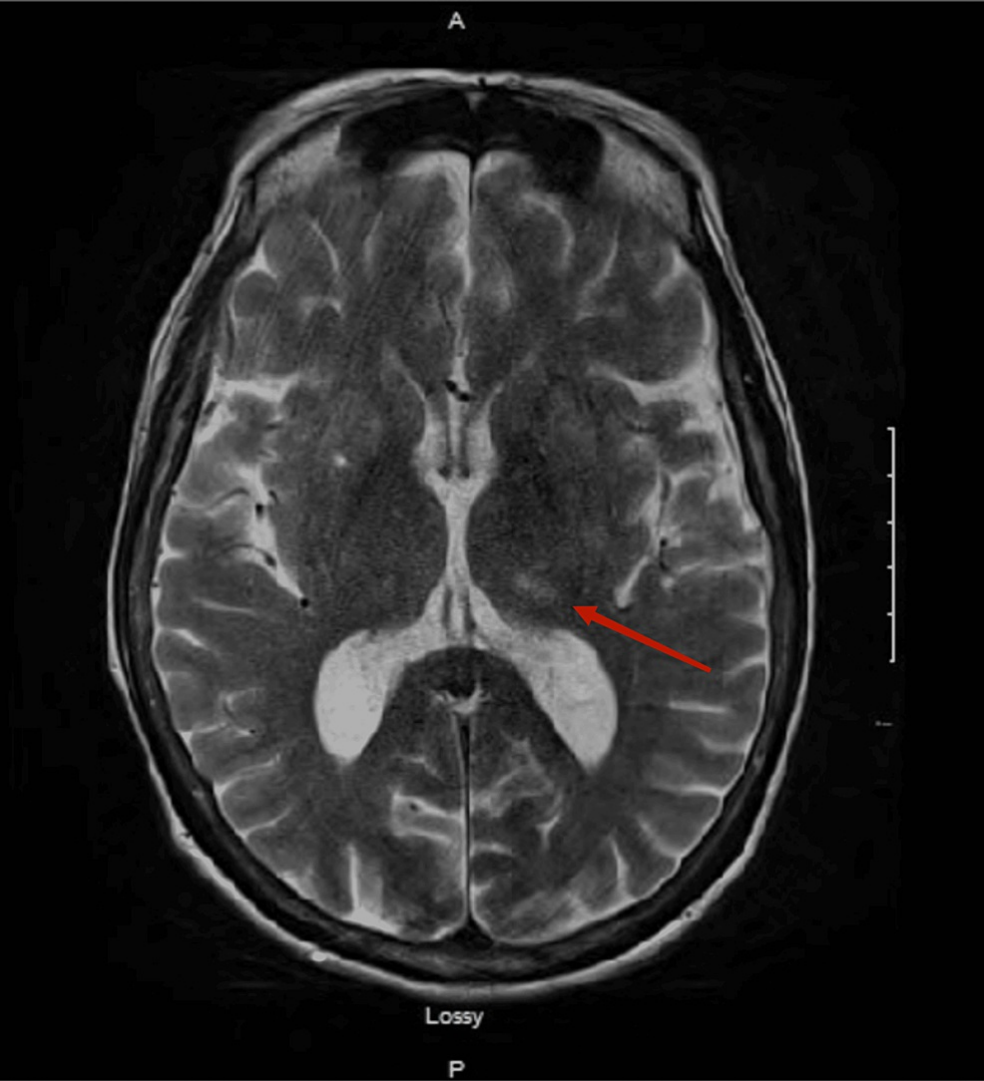
MRI brain and brainstem without contrast Increased T2 signal within bilateral thalami (left > right)

**Figure 2:**
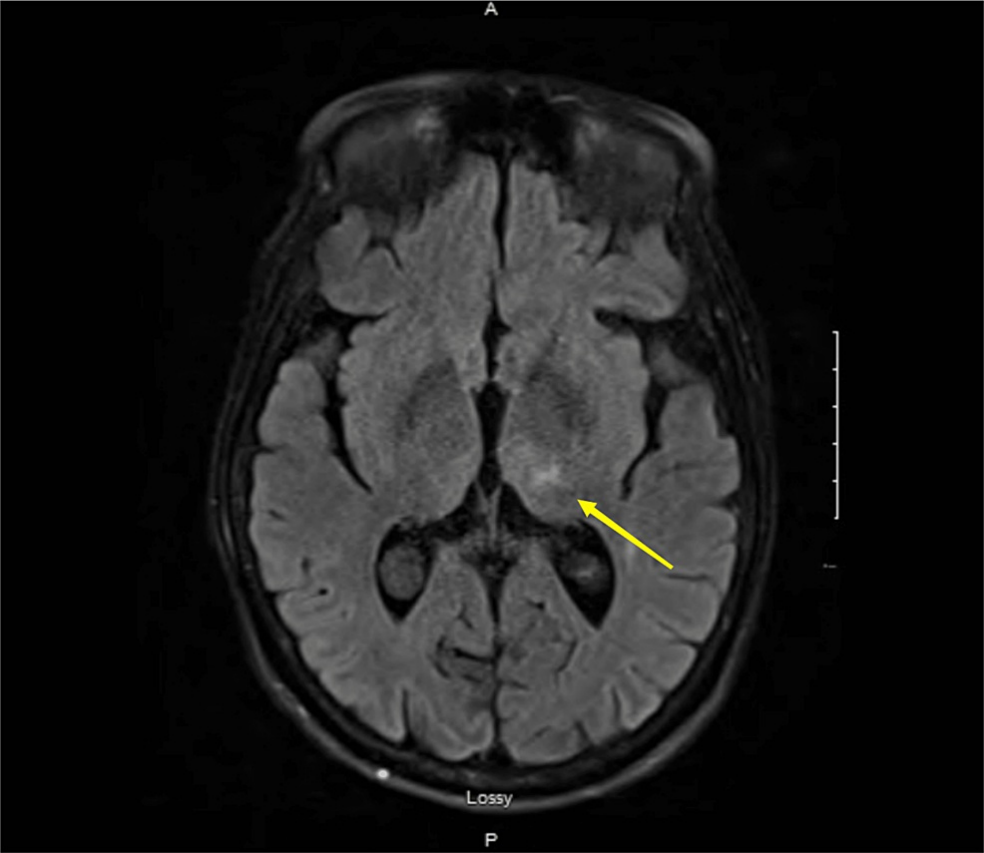
T2-weighted FLAIR images Bilateral thalamic hyperintensities (left > right) FLAIR: Fluid attenuated inversion recovery

A lumbar puncture was not performed due to the patient's complicated history of lumbar spinal surgery. An MRI of the brain revealed bilateral thalamic T2 signal intensity changes (with the left side greater than the right), suggestive of prior infarction or WE (Figures 1, 2).

A high dose of intravenous thiamine was promptly initiated (500 mg every eight hours). Remarkably, by the next morning, the patient demonstrated marked improvement in her cognitive state. Upon physical examination, she was alert and oriented to person, place, time, and situation (AAO x4). The significant cognitive improvement following the high-dose thiamine treatment strongly suggested WE. A comprehensive thiamine regimen was continued: 500 mg IV every eight hours for two days, followed by 250 mg IV every 12 hours for two days, and then 250 mg IV daily for three days. Subsequently, the patient was transitioned to oral thiamine therapy.

Post-recovery, the patient shared important details: no alcohol consumption but a compromised diet over the past year. Her daily intake primarily consisted of 1-2 reduced-portion frozen meals, leading to a weight loss of over 20 lbs within one year.

Day 12

After 12 days of thiamine treatment, the patient made a full recovery with no residual symptoms and was subsequently discharged.

## Discussion

WE is the presence of neurological symptoms caused by biochemical lesions of the central nervous system following the depletion of B-vitamin reserves, especially thiamine (vitamin B1). The condition is part of a spectrum of thiamine deficiency disorders, which encompasses various forms of beriberi and alcoholic Korsakoff syndrome. The concomitant presence of WE with alcoholic Korsakoff syndrome is known as Wernicke-Korsakoff syndrome [1-5].

Classically, WE is characterized by a triad of symptoms: ophthalmoplegia, ataxia, and confusion. However, only about 10% of patients exhibit this complete triad, with some manifesting additional or varied clinical features. While it is commonly regarded as a condition peculiar to malnourished people with alcohol misuse, it can be caused by a variety of diseases. Timely intervention with thiamine supplementation often improves symptoms and even results in full resolution, particularly when alcohol misuse is not the primary etiology. Concurrent replenishment of other depleted nutrients might be necessary, contingent on the specific underlying cause. Prompt and appropriate management is pivotal to preventing symptom exacerbation, as emphasized in numerous medical studies [6-8].

Thiamine serves as a cofactor for pyruvate and α-ketoglutarate dehydrogenase reactions, which produce adenosine triphosphate. In its absence, intracellular energy deficits would increase, eventually leading to cell death [7-10]. To prevent this, the body can store up to 30-50 mg of thiamine which necessitates only 1-2 mg daily for optimal physiological functions. Although alcohol intake may hasten thiamine depletion by driving the pyruvate dehydrogenase reaction, malnutrition alone would take at least 4-6 weeks to deplete intrinsic thiamine reserves. Acute hypoglycemic episodes predominantly seen in diabetics, are influenced by factors such as inadequate nutrition, increased physical exertion, drug interactions, or sepsis. These patients would not require routine thiamine loading unless they are at risk for thiamine deficiency. The administration of thiamine should always precede glucose to prevent the precipitation of acute WE is unfounded [8-13].

This is a case where a patient presented with AMS attributed to infection and AKI. WE, a crucial differential diagnosis, was not initially identified due to the simultaneous administration of thiamine with glucose and the absence of a history of alcohol use. Following her recovery, the patient revealed more pertinent details, including her malnutrition status resulting in a weight loss of 20 pounds within one year. These additional factors further support the diagnosis of WE.

Ataxia, typically characteristic of WE, was not described in this case due to her complicated picture of sepsis and AKI. However, MRI findings of thalamic T2 signal intensity changes gave us the clue. WE would have been more suggestive had the AMS developed after prolonged exposure to dextrose and prior to thiamine administration. However, this patient already presented with AMS on admission and received simultaneous administration of one amp of dextrose and 100mg thiamine. The onset of her WE indicates that her thiamine reserves had been critically low for a long time [13-15].

## Conclusions

This case underscores the critical importance of recognizing the risk of WE in patients with a history of chronic malnutrition. It is essential to be vigilant because the development of WE can occur even when thiamine is coadministered with glucose. In this context, MRI findings showing thalamic T2 signal intensity changes are instrumental in confirming the diagnosis. Early detection and prompt treatment are vital to prevent potentially severe neurological complications associated with this condition.
